# Association between diabetes mellitus and health-related quality of life among patients with chronic kidney disease: results from the Chinese Cohort Study of Chronic Kidney Disease (C-STRIDE)

**DOI:** 10.1186/s12955-020-01519-5

**Published:** 2020-08-03

**Authors:** Li Chen, Jinwei Wang, Xiaoyan Huang, Fang Wang, Wei Liang, Yan He, Yumei Liao, Luxia Zhang, Minghui Zhao, Zuying Xiong, on behalf of the C-STRIDE study group

**Affiliations:** 1 Renal Division, Peking University Shenzhen Hospital, Shenzhen, China; 2 Renal Division, Department of Medicine, Peking University First Hospital, Beijing, China; 3 Institute of Nephrology, Peking University, Beijing, China; 4 Key Laboratory of Renal Disease, Ministry of Health of China, Peking University, Beijing, China; 5 Key Laboratory of Chronic Kidney Disease Prevention and Treatment, Peking University, Beijing, China; 6 Center for Data Science in Health and Medicine, Peking University, Beijing, China

**Keywords:** Chronic kidney disease, Diabetes mellitus, Estimated glomerular filtration rate, Health-related quality of life

## Abstract

**Background:**

The prevalence of diabetes mellitus (DM) among patients with chronic kidney disease (CKD) has been increasing in recent years in China. This study aimed to evaluate the association between DM and health-related quality of life (HRQOL) in patients with CKD.

**Methods:**

In our study, participants with CKD stage 1 to 4 from 39 centers in China were screened and enrolled. The Kidney Disease Quality of Life (KDQOL™-36) questionnaire was used to assess HRQOL. Participants were divided into a diabetic group and a non-diabetic group. Demographic data, clinical data, and HRQOL scores were compared between the two groups. Multivariable robust regression was used to analyze the factors related to HRQOL in CKD patients.

**Results:**

A population of 2742 CKD patients was included in this study. CKD patients with DM were older and had lower education level, longer treatment periods and a higher prevalence of cardiovascular disease than CKD patients without DM (*P* < 0.05). HRQOL scores in the “symptoms and problems”, “effects of kidney disease”, and “SF-12 physical function” dimensions were significantly lower in the diabetic group than the non-diabetic group (86.88 ± 13.76 vs. 90.59 ± 10.75, 84.78 ± 14.86 vs. 87.28 ± 12.45, and 41.40 ± 9.77 vs. 45.40 ± 8.82, respectively, all *P <* 0.05). DM was negatively correlated with the symptoms and problems (regression coefficient for log transformed [175-score] = 0.010) and the SF-12 physical function dimension (regression coefficient = − 2.18) (all *P* < 0.05).

**Conclusion:**

HRQOL of diabetic patients with CKD was worse than that of non-diabetic patients with CKD. DM was an independent and negative factor affecting HRQOL in patients with CKD.

## Background

Chronic kidney disease (CKD) has become an important public health problem worldwide [1–4]. The first cross-sectional epidemiological survey of CKD in China showed that the prevalence of CKD in adults is 10.8% [5]. With the development of the national economy, the etiology spectrum of CKD is also changing in China. Diabetes mellitus (DM) has gradually replaced chronic glomerulonephritis as the main cause of renal function decline. In both 2010 and 2015, the proportion of urban CKD inpatients with DM exceeded that of urban CKD inpatients with glomerulonephritis [6]. In the Joint Asia Diabetes Evaluation (JADE) program, approximately 20% of the diabetics in China are diagnosed before the age of 40 years [7]. This means that more people will develop kidney damage during their lifetime. CKD and DM are both progressive diseases, with high morbidity, disability, and costs and a long survival period. A study of the Chinese Cohort Study of Chronic Kidney Disease (C-STRIDE) found that, compared with patients without DM, patients with DM have a higher prevalence of complications, such as hypertension, hyperlipidemia, anemia, hypoalbuminemia, and vascular disease [8].

Health-related quality of life (HRQOL) is one of the major goals of modern medicine, which is appropriate for CKD and DM patients receiving long-term treatment and care for their progressive and complex conditions. Assessing HRQOL and related factors in patients with CKD and DM is important to guide the educational direction of health and appropriate individualized care. Although DM has become the main cause of CKD, the HRQOL of Chinese patients with both CKD and DM is still unknown. Our study was designed to describe HRQOL in patients with CKD and DM and to analyze the association between DM and HRQOL in patients with CKD in stages 1 to 4 from C-STRIDE. Early identification and intervention of glucose metabolism problems in patients with CKD by clinicians will contribute to their high quality of life.

## Methods

### Study population

The C-STRIDE study enrolled participants with CKD in stages 1 to 4 between November 2011 and June 2017; this study was carried out in 39 clinical centers located in 28 provinces in China. The specific design has been published in a previous paper [9]. The C-STRIDE study is an ongoing prospective cohort enrolling patients with CKD in stages 1 to 4. In the current study, we used the data of 3541 participants, who were enrolled by June 2016. A total of 2742 of the C-STRIDE participants had completed the questionnaires, and their relevant records were preserved. Those with missing values for fasting plasma glucose (FPG) (*n* = 514) or the Kidney Disease Quality of Life (KDQOLTM-36) questionnaire (*n* = 326) were excluded from the analysis. In our study, the participants were divided into a diabetic group and a non-diabetic group. Demographic data, specific clinical data, and associated HRQOL scores were compared between the two groups. In the stratified analysis, the participants were divided into two groups according to their eGFR: CKD stage 1 to 2, with eGFR≥60 ml/min/1.73 m^2^, and CKD stage 3 to 4, with an eGFR range of 15-59 ml/min/1.73m^2^.

Height, weight, waist circumference, and hip circumference were measured in all patients. Specific biochemical markers were evaluated, such as serum creatinine (SCr), blood urea nitrogen (BUN), ACR, and FPG, as baseline data. In addition to the KDQOL™-36 scale, the questionnaire also contained general information, annual income, health-care costs, lifestyle, chronic comorbidities, complications, and medications, etc. Data collection was performed by trained staffs.

### Definitions

CKD was defined as either kidney damage or a decreased eGFR of less than 60 ml/min/1.73 m^2^ for at least 3 months. CKD was divided into 5 stages according to the KDIGO criteria [10]. The eGFR level was calculated by the modified GFR estimating equation in China if SCr was measured by Jaffe’s kinetic method [11] or by the CKD-EPI creatinine equation if SCr was measured by the Roche enzymatic method [12]. The diagnosis of DM followed the criteria established by the American Diabetes Association [13], alternatively, it was defined by a history of diabetes and/or the reported use of antidiabetic agents. Diabetic kidney disease (DKD) is a type of kidney disease caused by diabetes. Other causes of CKD are hypertension, metabolic disorders, interstitial nephritis and primary glomerulonephritis, etc. Patients with DKD were identified according to the following criteria: 15 (ml/min/1.73 m^2^) ≤ estimated glomerular filtration rate (eGFR) < 60 (ml/min/1.73 m^2^) or eGFR≥60 (ml/min/1.73 m^2^) with 24-h urinary protein≥3.5 g, urinary albumin/creatinine ratio (ACR) ≥ 2000 (mg/g), or corresponding values on the urine dipstick or urinary protein creatinine ratio (PCR) [9]. Glomerulonephritis patients were those with an eGFR ≥15 (ml/min/1.73 m^2^), and the inclusion criteria for patients with other causes of CKD were set as 15 (ml/min/1.73 m^2^) ≤ eGFR< 60 (ml/min/1.73 m^2^); patients with non-glomerulonephritis were identified according to the following criteria: 15 (ml/min/1.73 m^2^) ≤ eGFR< 60 (ml/min/1.73 m^2^) or eGFR≥60 (ml/min/1.73 m^2^) [9]. Smoking patients included current and former smokers. Education level was divided into a high school or above group and a below high school group. Economic burden was defined as the percentage of total household income spent on treatment, and heavy economic burden was defined as the cost of treatment accounting for more than 70% of total household income. Body weight was collected and categorized according to the Chinese body mass index (BMI) classification criteria published in 2003: underweight < 18.5 kg/m^2^, normal weight 18.5–23.9 kg/m^2^, overweight 24.0–27.9 kg/m^2^, or obese ≥28 kg/m^2^. ACR was calculated by spot urine albumin and creatinine. FPG was defined as non-caloric food intake for at least 8 h. Cardiovascular disease (CVD) was defined as the history of myocardial infarction, congestive heart failure, or arrhythmia events (recovery from cardiac arrest and ventricular fibrillation, ventricular tachycardia, atrial fibrillation or flutter and atrioventricular block).

### Quality of life assessment

The Chinese version of the KDQOL™-36 questionnaire was used to assess HRQOL in CKD patients; this was proven to be a simple, effective, and credible instrument [14–16]. The Center for Medicare Services in the United States mandated the KDQOL-36 questionnaire as a routine instrument to assess HRQOL in dialysis patients. The scale includes 5 dimensions: symptoms and problems (S), effects of kidney disease (E), burden of kidney disease (B), SF-12 physical function (PCS), and SF-12 mental function (MCS). PCS is a versatile tool to assess HRQOL in patients and in the healthy population [17]. The original scores were linearly converted to a range of 0–100. A high score represents better HRQOL. A KDQOLTM-36 questionnaire was self-reported by the participant after verbal guidance from the trained study staffs.

### Statistical analysis

Data analysis was performed by investigators who had not participated in the screening. All statistical analyses were performed using Statistical Analysis System (version 9.4, SAS Institute, Cary, NC). Continuous data are presented as the mean ± standard deviation (SD) or median (interquartile range, IQR). The *t*-test was used for the continuous variables conforming to the Gaussian distribution, while the Mann-Whitney U test was used for the continuous variables conforming to a skewed distribution. Normality for the distribution of each variable was tested by calculating skewness and kurtosis. The absolute value of either parameter greater than 3 was considered as the indication for a skewed distribution. The variables in skewed distribution, such as ACR, were under a logarithmic transformation before being used in the linear regression model. Categorical variables were presented as proportions and examined by the chi-square test. Univariate linear analysis was used to reveal the relationship between different variables and the dimensions of HRQOL. The homoscedasticity for each variable was tested by using the White test. If evidence of heteroscedasticity was detected, robust regression with the estimation method of M would be used. Multivariable regression analysis was performed to identify factors associated with HRQOL. Covariates in the multivariable regression were selected from those with statistical significance in the univariate analysis. However, FPG was not included in the multivariable regression, because the variable was a component to define DM and may introduce high collinearity in the model. Covariates included in the multivariable regression models were age, sex (male vs. female), education (≥high school vs. <high school), treatment period (≥1 year vs. < 1 year), economic burden (≥ 70% vs. < 70%), smoking (yes vs. no), BMI, ACR (log transformed), DM (yes vs. no), and CVD (yes vs. no). Collinearity was tested by calculating variance inflation factors. Similarly, given some independent variables violating homoscedasticity, robust regression was employed in the multivariable analysis. Finally, we examined the association between diabetes and HRQOL stratified by CKD stages. A *P* value < 0.05 (2-sided) was considered statistically significant. Missing values were filled with mean/median values for continuous variables and classified as a separate category for categorical variables before including the variables in the multivariable regression analysis. Sensitivity analysis was conducted by including the patients with complete data.

## Results

### Characteristics of the population

Altogether, 2742 CKD patients were included in the current analysis, with a mean age of 48.80 ± 13.66 and a mean eGFR of 54.88 ± 30.55 ml/min/1.73 m^2^; 59.59% were males, 40.41% were females, and 22.2% had DM. The mean scores or median scores of the 5 HRQOL dimensions were as follows: symptoms and problems = 89.76 ± 11.59, effects of kidney disease = 86.72 ± 13.07, burden of kidney disease = 51.30 ± 28.20, SF-12 physical function = 44.50 ± 9.19, and SF-12 mental function = 50.53 ± 9.07. Demographic and laboratory data of the diabetic and non-diabetic groups are presented in Table 1. The 799 patients excluded from the analysis were on average 2 years older, and had lower levels of ACR, eGFR and proportion of CVD, compared with those included in the analysis (Supplementary Table 1). The mean age of the diabetic patients was 57.0 ± 10.8 years, and 62.95% were male. Compared to the non-diabetic group, the diabetic population was older and had lower education, longer treatment period, more smoking, greater BMI, more albuminuria and lower eGFR values. More people had CVD in the diabetic group compared to the non-diabetic group. There was no significant difference in economic burden between the two groups.

**Table 1:** Comparison of general characteristics and clinic parameters of diabetic and non-diabetic groups in Chinese CKD patients

Variable	Category	Total	Diabetics	Non-diabetics	*P-*value
		(*n* = 2742)	(*n* = 610)	(*n* = 2132)	
Age (year) ^*^		48.80 ± 13.66	56.86 ± 10.82	46.55 ± 13.49	< 0.001^**^
Sex ^$^	Male	1634 (59.59%)	384 (62.95%)	1250 (58.63%)	0.055
	Female	1108 (40.41%)	226 (37.05%)	882 (41.37%)	
Marriage ^$^	Married	2350 (89.05%)	538 (92.44%)	1812 (88.09%)	0.003^**^
	Unmarried	289 (10.95%)	44 (7.56%)	245 (11.91%)	
Race ^$^	Ethnic Han	2526 (92.53%)	565 (92.93%)	1961 (92.41%)	0.67
	Others	204 (7.47%)	43 (7.07%)	161 (7.59%)	
Education ^$^	high school or above	832 (30.62%)	132 (21.93%)	700 (33.10%)	< 0.001^**^
	below high school	1885 (69.38%)	470 (78.07%)	1415 (66.90%)	
Treatment period ^$^	≥1 year	1434 (57.45%)	356 (64.03%)	1078 (55.57%)	< 0.001^**^
	<1 year	1062 (42.55%)	200 (35.97%)	862 (44.43%)	
Smoking history ^$^	Smoked	1021 (37.00%)	276 (46.15%)	745 (35.73%)	< 0.001^**^
	Never smoked	1662 (63.00%)	322 (53.85%)	1340 (64.27%)	
Economic burden ^$^	≥70%	551 (21.53%)	127 (22.48%)	424 (21.26%)	0.535
	<70%	2008 (78.47%)	438 (77.52%)	1570 (78.74%)	
BMI (kg/m^2^) ^*^		24.58 ± 3.54	25.52 ± 3.42	24.31 ± 3.53	< 0.001**
ACR (mg/g) ^#^		342.25 (74.16–901.58)	483.93 (94.87–1350.77)	312.41 (71.65–798.31)	< 0.001^**^
FPG (mmol/L) ^#^		4.96 (4.46–5.66)	6.72 (5.34–8.10)	4.81 (4.38–5.26)	< 0.001^**^
eGFR (ml/min/1.73m^2^) ^*^		54.88 ± 30.55	45.50 ± 23.92	57.56 ± 31.69	< 0.001^**^
CVD ^$^	Yes	343 (12.67%)	133 (21.88%)	210 (10.00%)	< 0.001^**^
	No	2366 (87.33%)	475 (78.13%)	1891 (90.00%)	
Symptoms and problems (S) ^#^		89.76 ± 11.59	86.88 ± 13.76	90.59 ± 10.75	< 0.001^**^
Effects of kidney disease (E) ^*^		86.72 ± 13.07	84.78 ± 14.86	87.28 ± 12.45	< 0·001^**^
Burden of kidney disease (B) ^*^		51.30 ± 28.20	49.85 ± 27.41	51.72 ± 28.41	0·148
SF-12 Physical Function (PCS) ^*^		44.50 ± 9.19	41.40 ± 9.77	45.39 ± 8.82	< 0·001^**^
SF-12 Mental Function (MCS) ^*^		50.53 ± 9.07	50.15 ± 9.82	50.64 ± 8.85	0.263

Abbreviations: *BMI* Body-mass index, *FPG* Fasting plasma glucose, *eGFR* Estimated glomerular filtration rate, *ACR* Albumin/creatinine ratio, *CVD* Cardiovascular disease

Number of missing: Age: 0; Sex: 0; Marriage: 103; Race: 12; Education: 25; Treatment period: 246; Smoking: 59; Economical burden: 183; BMI: 349; ACR: 370; FPG: 0; eGFR: 0; Cardiovascular disease: 33

Note 1: ^*^ The variables are numerical and statistics are Mean (Standard deviation), *P*-value calculated based on *t* test

Note 2: ^#^ The variables are numerical and statistics are Median (Interquartile range), *P*-value calculated based on Wilcoxon test

Note 3: ^$^ The variables are categorical and statistics are Frequency (Percentage), *P*-value calculated based on Chi-square test

Note 4: The denominator of percentage is number of the variable without missing values

Note 5: ^**^ Statistically significant at 0.05

### Quality of life

The scores in the “symptoms and problems”, “effects of kidney disease” and “SF-12 physical function” dimensions were statistically lower in the diabetic group than in the non-diabetic group (86.88 ± 13.76 vs. 90.59 ± 10.75, 84.78 ± 14.86 vs. 87.28 ± 12.45, and 41.40 ± 9.77 vs. 45.40 ± 8.82, respectively, *P* < 0.05). The other dimensions were not significantly different between the two groups, but the score in the diabetic group was lower than that in the non-diabetic group (Table 1). In the White tests, the standard errors of sex, economic burden, eGFR, CVD, logarithm transformed ACR and DM showed evidence of heteroscedasticity in the analysis for every dimension of HRQOL (*P* values< 0.05). According to univariate analysis, DM was associated with all five HRQOL dimensions (*P* < 0.05). Other factors were related to the 3 dimensions. Older age, lower education, longer treatment period, lower eGFR, history of CVD, higher levels of FPG and ACR were associated with lower scores. Multivariable regression analysis revealed that DM was negatively related to dimension S and PCS (regression coefficient: 0.010 for ln [175-score of S] and − 2.18 for PCS, *P* < 0.05), although the absolute value of the regression coefficient decreased after adjusting for other factors (Table 2). There is little possibility that collinearities could have influenced the estimation of regression coefficients, given that all variance inflation factors were less than 2 in the multivariable analysis for each dimension of HRQOL. The results of sensitivity analysis using those with complete data showed consistent results with the main analysis (Supplementary Table 2).

**Table 2:** The linear regression between DM and KDQOL™-36 scales

Regression coefficient	Log transformed symptoms and problems (S)^**^		Effects of kidney disease (E)		Burden of kidney disease (B)		SF-12 Physical Function (PCS)		SF-12 Mental Function (MCS)	
	univariate regression	multivariable regression^a^	univariate regression	multivariable regression^b^	univariate regression	multivariable regression^c^	univariate regression	multivariable regression^d^	univariate regression	multivariable regression^e^
Age (year)	0.0018^*^	0.0008^*^	− 0.071^*^	− 0.027	0.042		−0.15^*^	− 0.084^*^	− 0.0023	
Sex	−0.024^*^	− 0.022^*^	1.06^*^	0.86	5.54^*^	4.08^*^	1.55^*^	1.46^*^	0.81^*^	0.64
Marriage	0.0084		0.44		1.52		0.39		−0.29	
Race	−0.0011		−0.96		2.90		−0.72		1.10	
Education	−0.032^*^	−0.010^*^	1.13^*^	−0.60	6.20^*^	2.32	2.12^*^	0.50	0.83^*^	0.10
Treatment period	0.029^*^	0.015^*^	−1.56^*^	−0.70	−2.36^*^	− 1.05	− 1.76^*^	− 0.80^*^	− 0.14	
Smoking	− 0.0021		− 0.033		2.33^*^	0.49	−0.13		0.22	
Economic burden	0.045^*^	0.036^*^	−4.75^*^	−4.67^*^	−21.39^*^	−19.08^*^	− 4.48^*^	− 3.88^*^	− 3.12^*^	− 2.68^*^
BMI (kg/m^2^)	−0.00073		0.19^*^	0.20^*^	0.52^*^	0.39	0.064		0.17^*^	0.15^*^
FPG (mmol/L)	0.0089^*^		−0.66^*^		−0.93^*^		−0.78^*^		−0.059	
eGFR (ml/min/1.73 m^2^)	−0.00068^*^	−0.0003^*^	0.041^*^	0.021^*^	0.085^*^	0.048^*^	0.061^*^	0.026^*^	0.021^*^	0.012^*^
CVD	0.057^*^	0.040^*^	−2.93^*^	−2.36^*^	−2.25		−5.29^*^	−3.60^*^	−1.65^*^	−1.66^*^
**DM**	0.030^*^	0.010^*^	− 1.39^*^	−0.81	− 1.89^*^	− 1.08	−4.04^*^	−2.18^*^	− 0.22^*^	− 0.078
Log (ACR (mg/g))	0.0043^*^	0.0034^*^	−0.37^*^	− 0.31^*^	− 1.72^*^	− 1.12^*^	− 0.37^*^	−0.29^*^	− 0.35^*^	−0.30^*^

Note 1: ^*^ Statistically significant at 0.05

Note 2: ^**^ The log transformation was performed by the formula: ln (175-score of symptoms and problems)

Note 3: a Adjusted for age, sex, education, treatment period, economic burden, eGFR, CVD and Log (ACR)

Note 4: b Adjusted for age, sex, education, treatment period, economic burden, BMI, eGFR, CVD and Log (ACR)

Note 5: c Adjusted for sex, education, treatment period, smoking, economic burden, BMI, eGFR and Log (ACR)

Note 6: d Adjusted for age, sex, education, treatment period, economic burden, eGFR, CVD and Log (ACR)

Note 7: e Adjusted for sex, education, economic burden, BMI, eGFR, CVD and Log (ACR)

### Quality of life in the stratified analyses

In the stratified analysis, DM was associated with dimensions of E and PCS in the subgroup of CKD stages 1 to 2, while associated with S and PCS in CKD stages 3 to 4. Although multivariable adjustments attenuated the magnitude of association, the statistical significance was largely unchanged. The magnitude of association for the dimension of PCS was a little weaker in CKD stages 1 to 2 than that in CKD stages 3 to 4 with the regression coefficients of − 1.81 and − 2.42, respectively. (Table 3).

**Table 3:** The linear regression between diabetes and KDQOL™-36 scales stratified by CKD stages

KDQOL™-36 scales	CKD stage 1–2		CKD stage 3–4	
	(*n* = 979)		(*n* = 1763)	
	univariate regression	multivariable regression^**^	univariate regression	multivariable regression^**^
Log transformed symptoms and problems (S)^†^	0.015	−0.0027	0.029^*^	0.016^*^
Effects of kidney disease (E)	−1.95^*^	−2.11^*^	−0.71	−0.53
Burden of kidney disease (B)	−4.64	− 4.37	0.071	−0.36
SF-12 Physical Function (PCS)	−3.39^*^	−1.81^*^	− 3.53^*^	−2.42^*^
SF-12 Mental Function (MCS)	−1.08	−1.15	0.33	0.43

Abbreviations: *CKD* Chronic kidney disease

Note 1: ^*^Statistically significant at 0.05

Note 2: ^**^Covariates are the same as those used for each KDQOL™-36 scale in Table 2

Note 3: ^†^ The log transformation was performed by the formula: ln (175-score of symptoms and problems)

## Discussion

In this study, demographic data, clinical characteristics and HRQOL were compared between CKD patients (stages 1 to 4) with diabetes and without diabetes. Kidney injury was more severe in the diabetic group than in the non-diabetic group. HRQOL scores were significantly lower in the diabetic group in the “symptoms and problems”, “effects of kidney disease” and “SF-12 physical function” dimensions. Furthermore, DM was proved to be negatively associated with HRQOL in CKD patients in this study. Through our findings, we hope that clinicians will be aware of the relationship between DM and the quality of life in CKD patients, and try to improve their quality of life by appropriate intervention in DM.

Although there was no significant difference in the “burden of kidney disease” or “SF-12 mental function” dimensions, the diabetic group had lower mean scores on all dimension scales compared with the non-diabetic group, which suggested that diabetic patients with CKD suffered a more impaired HRQOL. According to univariate linear analysis, DM was associated with the “symptoms and problems”, “effects of kidney disease” and “SF-12 physical function” dimensions. When adjusted for several specific variables screened from univariate linear analysis, DM was still significantly associated with the dimensions of “symptoms and problems” and “SF-12 physical function”. Our findings were consistent with those of other countries. Two studies containing thousands of CKD participants revealed that DM was associated with low HRQOL in North America (*P* < 0.05) [18]. In the study conducted in North America in 2016, the differences in demographic and clinical characteristics between the diabetic and non-diabetic groups were similar to those of our study. However, unlike our study, the North American study revealed that the HRQOL scores of all dimensions were significantly different between the diabetic patients and the non-diabetic patients who were recruited from 3837 participants with CKD in stage 2 to 4 [18, 19]. This may be explained by the differences in the inclusion criteria, the national insurance policy and the humanistic values between the two studies. A study of 537 CKD participants in another country in Asia (Japan) found that HRQOL was impaired by the presence of DM, despite using a different HRQOL instrument [20]. In addition to assessing the current HRQOL of CKD patients, the “SF-12 physical function” dimension was also a factor associated with the survival rate and length of hospital stay. A previous study found that each 5-point increase in “SF-12 physical function” scores was related to a 10% increase in survival and 6% fewer hospital stay days [21]. Therefore, it can be speculated that the diabetic group may have lower survival rates and longer hospital stays than the non-diabetic group.

In the stratified analysis, we found that DM was negatively associated with the scores of HRQOL in both subgroups, but the dimensions and magnitude of correlation were different. In the stage of poor renal function and more complications (stages 3 to 4), the correlation between DM and the quality of life was strengthened in 2 dimensions of “symptoms and problems” and “SF-12 physical Function”. While the correlation between DM and the quality of life was weakened in 1 dimension, “effects of kidney disease”, which may be due to the enhanced negative effects of reduced eGFR and increased or aggravated complications of CKD on the quality of life. A study of 1186 CKD patients in North America also suggested that HRQOL in diabetic patients was lower than that in non-diabetic patients with CKD in stage 3 to 5 [19]. No matter what stage of kidney disease, DM always brings a poor life experience to this group of patients. Therefore, in the whole process of chronic disease management of patients with CKD, clinicians need to pay more attention to the management of DM, so as to optimize the long-term HRQOL. Especially when eGFR drops significantly, we should not neglect the management of DM.

DM and CKD are both global and public health problems [22]. It was estimated that there were 451 million diabetic patients worldwide in 2017 [23]. In China, there were no accurate data on the duration of DKD occurrence in diabetic patients, but it was known that Asians were more susceptible to DKD than Caucasians [24]. CKD and DM interact with each other, leading to deterioration of the kidney and other organs, such as the retina and the cardiovascular and nervous systems. Seventy-five percent of patients with DM die of CVD [25]. In addition, there is a high incidence of some tumors in these patients [26]. Activation of inflammatory mediators, inhibition of antioxidant defense mechanisms and insulin resistance were linked to the deterioration of kidney disease in patients with diabetes. Additionally, a previous study revealed that patients with CKD and DM suffer more complications and reduced uremia and volume load tolerance [8]. As a result, these patients had to bear more daily life restrictions and limited social activities, and they received dialysis earlier than non-diabetic patients. For these reasons, the diabetic patients with CKD have more negative factors impacting on their HRQOL [27]. Identifying and intervening early in DM will contribute to improving the HRQOL of these patients, despite the progressive deterioration of kidney function and complications in other organs.

### Limitations

Some limitations of the study should be considered. First, when interpreting the results for HRQOL, the personal assessments of health condition are strongly subjective and affected by non-healthy factors, such as cultural aspects and environmental changes. Second, a patient’s knowledge of their diagnosis may affect the perception of health in asymptomatic statements. Third, those excluded from the analysis due to missing values of FPG and scores of HRQOL had difference in some demographic and clinical characteristics, which may introduce selection bias to the study. Fourth, the small sample size in the stratified analysis may affect the results. Fifth, our study had a cross-sectional design. Therefore, the direction of the causal relationship cannot be established.

## Conclusion

In conclusion, diabetic patients had lower levels of HRQOL than non-diabetic patients among the Chinese CKD population. DM was an independent and negative factor affecting HRQOL in patients with CKD stages 1 to 4. Clinicians should identify DM early in patients with CKD, especially in those whose renal impairment is not attributable to diabetic nephropathy. In addition to controlling CKD and its complications, clinicians should also pay attention to the monitoring and treatment of diabetes in these patients. Developing health care programs for diabetic patients in the early stages of CKD may help patients to lead an active lifestyle and have a relatively high HRQOL in the future.

## Supplementary information

**Additional file 1: Table S1.** Characteristics of the included and excluded population.

**Additional file 2: Table S2.** The complete case analysis of linear regression between DM and KDQOLTM-36 scales.

## Data Availability

All data generated or analyzed during this study are included in this published article.

## Abbreviations

ACR: Albumin/creatinine ratio
B: Burden of kidney disease
BUN: Blood urea nitrogen
C-STRIDE: Chinese Cohort Study of Chronic Kidney Disease
CKD: Chronic kidney disease
CVD: Cardiovascular disease
DKD: Diabetic kidney disease
DM: Diabetes mellitus
E: Effects of kidney disease
eGFR: Estimated glomerular filtration rate
FPG: Fasting plasma glucose
HRQOL: Health-related quality of life
JADE: Joint Asia Diabetes Evaluation
KDQOL™-36: Kidney Disease Quality of Life
MCS: SF-12 mental function
PCR: Protein creatinine ratio
PCS: SF-12 physical function
S: Symptoms and problems
SCr: Serum creatinine
