# Supplementary material for: Association between diabetes mellitus and health-related quality of life among patients with chronic kidney disease: results from the Chinese Cohort Study of Chronic Kidney Disease (C-STRIDE)
Source: Health Qual Life Outcomes. 2020 Aug 3;18:266. doi: 10.1186/s12955-020-01519-5 (PMC7398214; doi:10.1186/s12955-020-01519-5)
Supplement: Supplementary file 1 — Additional file 1: Table S1. Characteristics of the included and excluded population. [file 12955_2020_1519_MOESM1_ESM.docx]

Supplementary table 1 Characteristics of the included and excluded population

| Variable | Category | Excluded population | Included population | P-value |
| --- | --- | --- | --- | --- |
|  |  | (n=799) | (n=2742) |  |
| Age (year) ^*^ |  | 50.77year) | 48.80year) p<0.001 |  |
| Sex ^$^ | Male | 465(58.20%) | 1634(59.59%) | 0.48 |
|  | Female | 334(41.80%) | 1108(40.41%) |  |
| Marriage ^$^ | Married | 509(63.70%) | 2350(89.05%) | <0.001 |
|  | Unmarried | 290(36.30%) | 289(10.95%) |  |
| Race ^$^ | Ethnic Han | 731(95.81%) | 2526(92.53%) | 0.001 |
|  | Others | 32(4.19%) | 204(7.47%) |  |
| Education ^$^ | high school or above | 187(30.21%) | 832(30.62%) | 0.84 |
|  | below high school | 432(69.79%) | 1885(69.38%) |  |
| Treatment period ^$^ | ≥reatmen240(59.70%) | 1434(57.45%) | 0.40 |  |
|  | ＜1 year | 162(40.30%) | 1062(42.55%) |  |
| Smoking history ^$^ | Smoked | 208(36.11%) | 1021(37.00%) | 0.38 |
|  | Never smoked | 368(63.89%) | 1662(63.00%) |  |
| Economic burden ^$^ | ≥cono119(20.95%) | 551(21.53%) | 0.76 |  |
|  | ＜70% | 449(79.05%) | 2008(78.47%) |  |
| BMI (kg/m2) ^*^ |  | 24.38kg/m2 | 24.58kg/m2)0.26 |  |
| ACR (mg/g) ^#^ |  | 277.00(52.50-772.76) | 342.25(74.16-901.58) | 0.006 |
| FPG (mmol/L) ^#^ |  | NA | 4.96(4.46-5.66) | NA |
| eGFR (ml/min/1.73m^2^) ^*^ |  | 49.25(ml/mi | 54.88(ml/min<0.001 |  |
| CVD ^$^ | Yes | 74(9.26%) | 343(12.67%) | 0.009 |
|  | No | 725(90.74%) | 2366(87.33%) |  |

Abbreviations: BMI = Body-mass index; FPG = fasting plasma glucose; eGFR = estimated glomerular filtration rate; ACR = albumin/creatinine ratio; NA=not available; CVD=cardiovascular disease.

Number of missing among the population excluded from the analysis: Race: 36; Education: 180; Treatment period: 397; Smoking: 223; Economical burden: 231; BMI: 292; ACR: 196.

Note 1: ^*^ The variables are numerical and statistics are Mean (Standard deviation), P-value calculated based on t test.

Note 2: ^#^ The variables are numerical and statistics are Median (Interquartile range), P-value calculated based on Wilcoxon test.

Note 3: ^$^ The variables are categorical and statistics are Frequency (Percentage), P-value calculated based on Chi-square test.

Note 4: The denominator of percentage is number of the variable without missing values.
